# Decarboxylation Study of Acidic Cannabinoids: A Novel Approach Using Ultra-High-Performance Supercritical Fluid Chromatography/Photodiode Array-Mass Spectrometry

**DOI:** 10.1089/can.2016.0020

**Published:** 2016-12-01

**Authors:** Mei Wang, Yan-Hong Wang, Bharathi Avula, Mohamed M. Radwan, Amira S. Wanas, John van Antwerp, Jon F. Parcher, Mahmoud A. ElSohly, Ikhlas A. Khan

**Affiliations:** ^1^ National Center for Natural Products Research, School of Pharmacy, University of Mississippi, University, Mississippi.; ^2^ Waters Corporation, Milford, Massachusetts.; ^3^ Department of Pharmaceutics and Drug Delivery, School of Pharmacy, University of Mississippi, University, Mississippi.; ^4^ Division of Pharmacognosy, Department of BioMolecular Science, School of Pharmacy, University of Mississippi, University, Mississippi.

**Keywords:** cannabinoids, *Cannabis sativa*, decarboxylation, kinetic analysis, UHPSFC/PDA-MS

## Abstract

**Introduction:** Decarboxylation is an important step for efficient production of the major active components in cannabis, for example, Δ^9^-tetrahydrocannabinol (Δ^9^-THC), cannabidiol (CBD), and cannabigerol (CBG). These cannabinoids do not occur in significant concentrations in cannabis but can be formed by decarboxylation of their corresponding acids, the predominant cannabinoids in the plant. Study of the kinetics of decarboxylation is of importance for phytocannabinoid isolation and dosage formulation for medical use. Efficient analytical methods are essential for simultaneous detection of both neutral and acidic cannabinoids.

**Methods:** *C. sativa* extracts were used for the studies. Decarboxylation conditions were examined at 80°C, 95°C, 110°C, 130°C, and 145°C for different times up to 60 min in a vacuum oven. An ultra-high performance supercritical fluid chromatography/photodiode array-mass spectrometry (UHPSFC/PDA-MS) method was used for the analysis of acidic and neutral cannabinoids before and after decarboxylation.

**Results:** Decarboxylation at different temperatures displayed an exponential relationship between concentration and time indicating a first-order or *pseudo*-first-order reaction. The rate constants for Δ^9^-tetrahydrocannabinolic acid-A (THCA-A) were twice those of the cannabidiolic acid (CBDA) and cannabigerolic acid (CBGA). Decarboxylation of THCA-A was forthright with no side reactions or by-products. Decarboxylation of CBDA and CBGA was not as straightforward due to the unexplained loss of reactants or products.

**Conclusion:** The reported UHPSFC/PDA-MS method provided consistent and sensitive analysis of phytocannabinoids and their decarboxylation products and degradants. The rate of change of acidic cannabinoid concentrations over time allowed for determination of rate constants. Variations of rate constants with temperature yielded values for reaction energy.

## Introduction

The plant *Cannabis sativa,* in the form of crude drugs, marijuana, hashish, or hash oil, is the most widely consumed and popular recreational/medicinal botanical drug product in the world.^1^ The legal status of cannabis varies significantly from state to state within the United States and also from country to country. As a result of the rampant use and confounding legal issues, there has been a significant case load increase seen in forensic laboratories. Therefore, cannabis is now one of the most thoroughly studied and analyzed plant materials. More than 100 cannabinoids have been isolated and identified in cannabis^2^ along with the primary psychoactive component, Δ^9^-tetrahydrocannabinol (Δ^9^-THC). In addition to Δ^9^-THC, there are other components of cannabis that have been shown to be medically beneficial. For example, cannabidiol (CBD) and cannabigerol (CBG) can moderate or influence the psychoactive effects of Δ^9^-THC.^3,4^ Studies of cannabis have also investigated the potential benefits of phytocannabinoids as anticancer, antiemetic, sedative, and palliative agents for several other disease states and symptoms.^3,5^

Efficient production of Δ^9^-THC, CBD, and CBG from cannabis is important for the development of dosage formulations to facilitate the successful medical use of cannabis. These neutral cannabinoids do not occur at significant concentrations in the plants. Cannabis synthesize primarily the carboxylic acid forms of Δ^9^-THC, CBD, and CBG, namely, Δ^9^-tetrahydrocannabinolic acid A (THCA-A), cannabidiolic acid (CBDA), and cannabigerolic acid (CBGA). These acidic cannabinoids are thermally unstable and can be decarboxylated when exposed to light or heat via smoking, baking, or refluxing. As a result, the requisite forensic analyses are usually expressed as the sum of the acidic and neutral forms of the cannabinoids. Reports also show that Δ^9^-THC itself readily oxidizes to cannabinol (CBN) with oxygen and light during the decarboxylation process.^6^

To understand the decarboxylation reactions that can occur with phytocannabinoids, efficient analytical methods are necessary to determine the concentration variations of decarboxylation reactants (acidic cannabinoids) and products (neutral cannabinoids) over time. Many analytical instruments have been applied to analyze cannabinoids in cannabis.^3,6,7^ Among them, gas chromatography (GC) and liquid chromatography (LC) are the most commonly used techniques.

GC is ideal in some ways for these low molecular weight (280–360) neutral cannabinoids. However, the labile acids cannot be analyzed by GC without decarboxylation or derivatization.^8^ Hewavitharana et al.^9^ reported the decarboxylation reaction conducted in a heated GC injection port and suggested that this process can provide a means of complete conversion of the acids to neutral cannabinoids. Likewise, Dussy et al.^6^ also studied the decarboxylation of pure Δ^9^-tetrahydrocannabinolic acid A (THCA-A), however, the generation of Δ^9^-THC was maximal at an intermediate temperature (225°C) but with only 65% conversion. At 300°C, a significant loss of Δ^9^-THC was observed, although no CBN, a possible oxidation product, was observed. Thus, the use of a GC injection port to convert THCA-A to Δ^9^-THC was not satisfactory under the experimental conditions of that particular study. In summary, GC analyses are complicated by the need for decarboxylation or derivatization of the acid cannabinoids before analysis. Moreover, both decarboxylation and derivatization techniques are subject to efficiency issues.

LC is another chromatographic technique commonly used for decarboxylation studies because it is capable of detecting both neutral and acidic cannabinoids. No decarboxylation or derivatization is necessary using this technique. Veress et al.^10^ studied the generation of Δ^9^-THC by heating dried extracts of cannabis over a range of temperature and time, and the products were analyzed by high-performance liquid chromatography/diode-array (HPLC/DAD). Maximum formation of Δ^9^-THC was observed in ∼5–10 min at 145°C followed by a significant loss at longer times possibly due to evaporation of Δ^9^-THC. Dussy et al.^6^ also heated pure THCA-A in an oven for a fixed time (15 min) at 120°C, 140°C, 160°C, and 180°C. The reaction products were also analyzed by HPLC/DAD. Conversion of THCA-A was complete at 160°C; however, formation of an oxidation product, CBN, was observed at 160°C and 180°C. Thus, the conversion of the acid to Δ^9^-THC was never perfectly complete without loss or degradation of starting material. In this study, the molar sum of Δ^9^-THC and THCA-A measured by HPLC/DAD was always higher than the total Δ^9^-THC measured by GC, indicating an incomplete decarboxylation reaction. More recently, Perrotin-Brunel et al.^11^ studied the kinetics and molecular modeling of the decarboxylation of THCA-A using HPLC. The proposed *pseudo*-first-order, acid catalyzed keto–enol mechanism for the decarboxylation process was found to be >95% efficient. The major problem with the HPLC/DAD analysis of acidic or neutral cannabinoids is the low molar absorptivity of these components, which results in relatively high limits of detection and restricts DAD detection to low wavelengths where there is often strong background absorbance from the eluant components, especially during gradient elution experiments. This problem can be overcome by using mass spectrometric detection.

Supercritical fluid chromatography (SFC) is a mild separation technique by which decarboxylation of the acid cannabinoids can be avoided.^12^ It is fast, cost-effective, and able to provide the resolution necessary to separate neutral and acidic cannabinoids simultaneously.^13^ Thus, ultra-high performance supercritical fluid chromatography (UHPSFC) with photodiode array (PDA) and mass spectrometry (MS) detections was used the first time to our knowledge to conduct a decarboxylation study of phytocannabinoids in a solvent extract of cannabis.

Most of the previously reported decarboxylation results emphasized only the conversion of THCA-A to Δ^9^-THC. In the current studies, decarboxylation studies of three acidic cannabinoids, namely, THCA-A, CBDA, and CBGA, were carried out over a range of temperature and time to determine the most appropriate conditions for complete decarboxylation. Beside the neutral and acidic cannabinoids from decarboxylation reaction, the possible oxidation product (CBN), the isomerization product Δ^8^-tetrahydrocannabinol (Δ^8^-THC), and tetrahydrocannabivarin (THCV) were also quantified simultaneously. In addition, the kinetic analysis, including the determination of decarboxylation reaction rate constants and reaction energies, was conducted based on the decrease in acidic cannabinoid concentrations over a range of time.

## Materials and Methods

### Materials and reagents

Optima-grade isopropanol and acetonitrile were purchased from Fisher Scientific. Deionized water was generated by the Millipore Milli-Q water purification system. Regular-grade carbon dioxide was obtained from New Air.

**FIG. 1.**
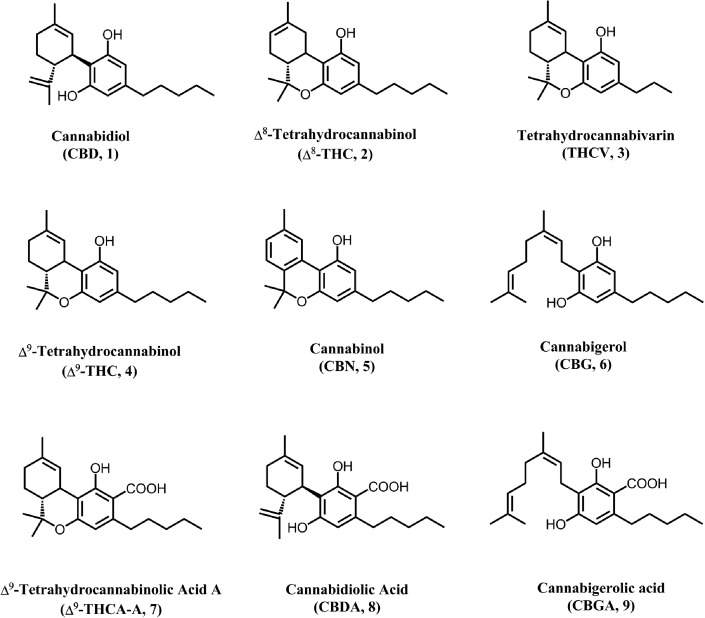
Structures of major cannabinoids in *Cannabis sativa.*

Nine cannabinoid reference standards, namely, CBD, Δ^8^-THC, THCV, Δ^9^-THC, CBN, CBG, THCA-A, CBDA, and CBGA, were isolated in-house at The National Center for Natural Products Research, University of Mississippi, from cannabis plant materials (structures are shown in Fig. 1). The identity and purity of the isolated standards were established by infrared spectroscopy, nuclear magnetic resonance, and liquid chromatography/quadrupole time-of-flight.

### Sample information

The extracts of *C. sativa* flowering buds, sample C-9, were used to conduct the decarboxylation studies. The sample was obtained from the supply of materials provided to “The University of Mississippi NIDA Marijuana Project” as part of the “Cannabis Potency Monitoring Program.” Plants were grown from *C. sativa* cuttings, and then the whole buds of mature female plants were harvested, air-dried, manicured, packed in barrels, and stored at low temperature (−24°C). The plant was authenticated by Dr. Suamn Chandra from the University of Mississippi. The extraction procedure involved maceration of 20 g powdered plant material in methylene chloride for 24 h (250 mL×2 times). The extraction solution was then evaporated under vacuum to dryness yielding 1.9 g of extract.

### UHPSFC/PDA-MS analysis

The UHPSFC/PDA-MS method was described previously.^14^ Briefly, the analysis was conducted on a Waters ACQUITY UPC^2^ system equipped with a photodiode array detector. The column was a Waters ACQUITY UPC^2^ BEH 2-EP column (150×3.0 mm I.D., 1.7 μm). Data acquisition was performed with MassLynx (4.1) software. Mobile phases consisted of CO_2_ (A) and isopropanol:acetonitrile (80:20) with 1% water (B). The gradient conditions were as follows: 4.0% B to 9.0% B in 4.5 min, and then to 30.0% B in the next 2.5 min (hold 3 min). The system was re-equilibrated for 6.5 min before the next injection. The flow rate was 1.4 mL/min. The injection volume was 1.0 μL. The column and autosampler temperatures were maintained at 30°C and 10°C, respectively. The UV wavelength was set to scan from 190 to 400 nm, and 220 nm was used for the quantification.

The MS data were acquired on a Waters ACQUITY single quadrupole mass spectrometer equipped with an electrospray ionization source operating at 150°C in scan mode from 100 to 800 amu for both positive and negative. The capillary voltage was 4.5 kV and cone voltage was 40 V. Nitrogen was used as desolvation and cone gas at flow rates of 500 and 50 L/h, respectively. The desolvation temperature was 400°C. The make-up flow composed of methanol with 8 mM ammonium formate and 0.5% formic acid was delivered at a flow rate of 0.6 mL/min. The active back pressure regulator pressure was 1500 psi.

### Sample preparation and decarboxylation reactions of *C. sativa* extract

For the decarboxylation reactions, separate vials were used for each experiment. The vial containing 3.0 mg/mL extracts dissolved in acetonitrile:methanol (80:20) was dried using a SpeedVac concentrator. An individual vial was placed in an oven for a fixed time at a fixed temperature for the decarboxylation reactions. A vacuum oven was used to eliminate oxygen and light that could possibly produce decomposition of Δ^9^-THC to CBN. Conditions for decarboxylation were examined at 80°C, 95°C, 110°C, 130°C, and 145°C for different times up to 60 min to determine the most appropriate experimental condition. After decarboxylation, the extracts were redissolved in acetonitrile:methanol (80:20) to give 1.0 mg/mL solutions for the UHPSFC/PDA-MS analysis. Duplicate samples were tested for each reaction.

## Results and Discussion

### Decarboxylation studies

**FIG. 2.**
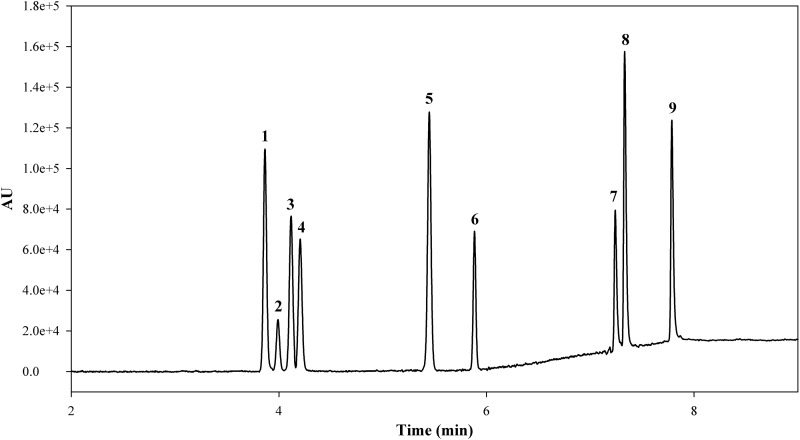
UHPSFC/PDA (220 nm) chromatogram of a mixture of cannabinoid standards. Peak assignment: (1) CBD, (2) Δ^8^-THC, (3) THCV, (4) Δ^9^-THC, (5) CBN, (6) CBG, (7) THCA-A, (8) CBDA, (9) CBGA. CBD, cannabidiol; CBDA, cannabidiolic acid; CBG, cannabigerol; CBGA, cannabigerolic acid; CBN, cannabinol; THC, tetrahydrocannabinol; THCA-A, tetrahydrocannabinolic acid-A; THCV, tetrahydrocannabivarin; UHPSFC, ultra-high-performance supercritical fluid chromatography.

A previously developed and validated UHPSFC/PDA-MS method^14^ was used for the analysis of cannabinoids from decarboxylation. The PDA (220 nm) data were used for the quantification. The calibration plots for all the analyzed cannabinoid standards were linear over the concentration range of 5.0–1000.0 μg/mL with the correlation coefficients (*R^2^*) > 0.994. The MS data were used for the compound identification and chromatographic peak purity check purpose. In this study, the nine cannabinoids (CBD, Δ^8^-THC, THCV, Δ^9^-THC, CBN, CBG, THCA-A, CBDA, and CBGA) were quantitatively determined before and after cannabis extracts were heated in a vacuum oven at 80°C, 95°C, 110°C, 130°C, and 145°C for up to 60 min. The full results of the experiments are given in Supplementary Table S1 in the Supplementary Data. Figure 2 shows the chromatogram of the nine major cannabinoids with PDA detection at 220 nm. The standards were well resolved with an elution order of neutral cannabinoids eluting before their precursor acids. This order is reversed from that observed for HPLC systems.^13^ Therefore, SFC analysis can serve as orthogonal methods to HPLC/GC and provide different relative retentions of peaks that are needed to ensure full characterization and confirmation analysis of cannabis.

#### Δ^9^-Tetrahydrocannabinolic acid A

**FIG. 3.**
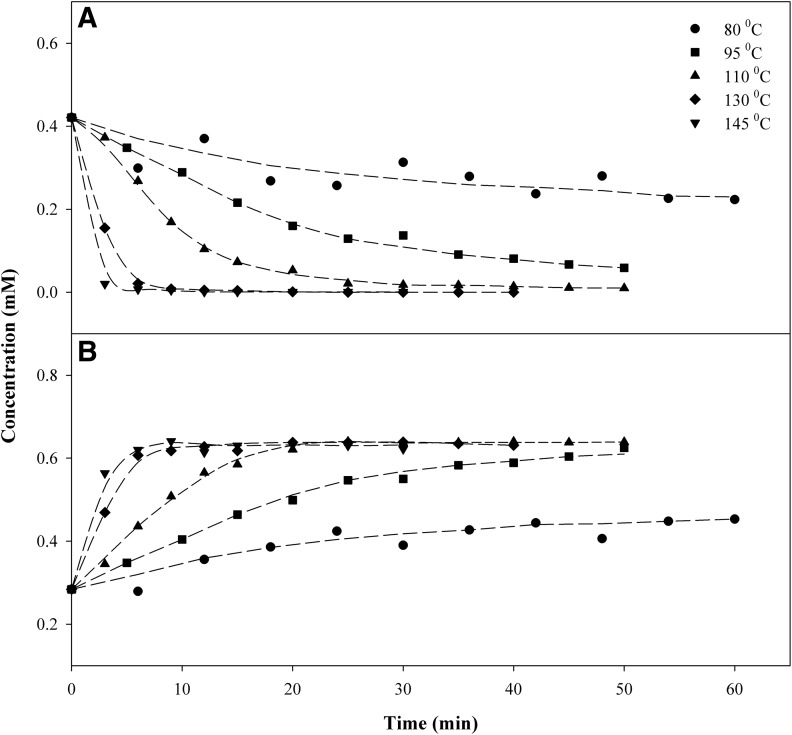
Concentration (mM) of **(A)** THCA-A and **(B)** Δ^9^-THC as a function of time and temperature.

**FIG. 4.**
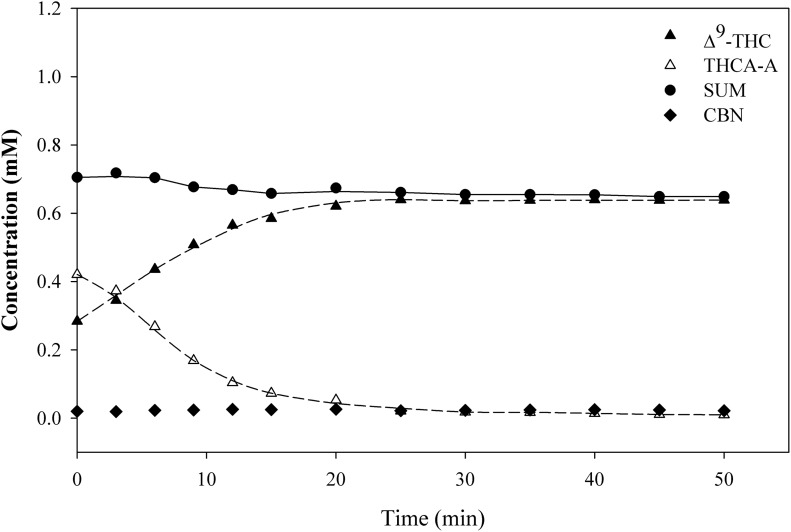
Experimental results for THCA-A, Δ^9^-THC and CBN at 110°C.

Figure 3 shows the results of the decarboxylation reactions for THCA-A→Δ^9^-THC. At temperatures lower than 100°C, the reaction did not reach completion within 60 min. At higher temperatures, the concentration of THCA-A approached zero in 30, 9, and 6 min at 110°C, 130°C, and 145°C, respectively. The stoichiometry of the reaction is shown in Figure 4, where the concentrations of Δ^9^-THC and THCA-A and their sum are plotted as a function of time. The results indicate complete conversion of THCA-A→Δ^9^-THC. The sum of the molar concentration of THCA-A and Δ^9^-THC decreased slightly after the decarboxylation, the relative loss is given in Table 1. Figure 4 also shows the data for CBN, which is a possible oxidation product of Δ^9^-THC. In this case, heating in the dark and in the absence of oxygen (vacuum oven) did not result in any significant oxidation of Δ^9^-THC to CBN. Finally, decarboxylation studies with *pure* THCA-A showed clearly that Δ^9^-THC was the only decomposition product observed after heating at 110°C for 40 min.

**Table 1.** **The Relative Loss for the Total Molar Concentration (Sum of Acidic Reactants and Neutral Products) Upon Completion of Decarboxylation**

Decarboxylation reaction	Form	Temperature (°C)	Relative loss in total molar concentration (%)
THCA-A→THC	Extracts	110	7.94
CBDA→CBD	Extracts	110	18.05
CBDA→CBD	Extracts	130	25.2
CBGA→CBG	Extracts	110	52.67
CBDA→CBD	Pure standard	110	13.75

CBD, cannabidiol; CBDA, cannabidiolic acid; CBG, cannabigerol; CBGA, cannabigerolic acid; THC, tetrahydrocannabinol; THCA-A, tetrahydrocannabinolic acid-A.

#### Cannabidiolic acid

**FIG. 5.**
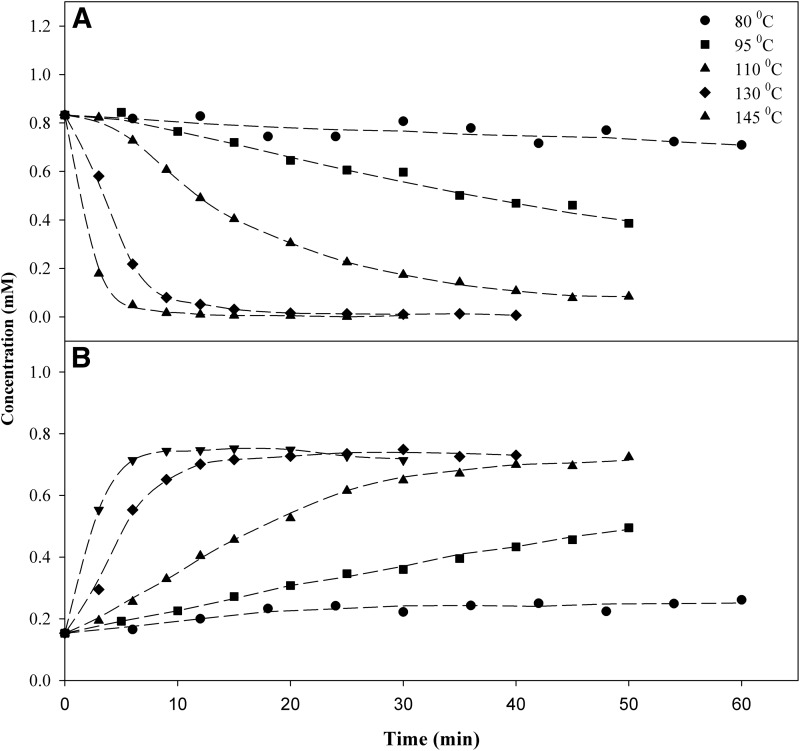
Concentration (mM) of **(A)** CBDA and **(B)** CBD as a function of time and temperature.

**FIG. 6.**
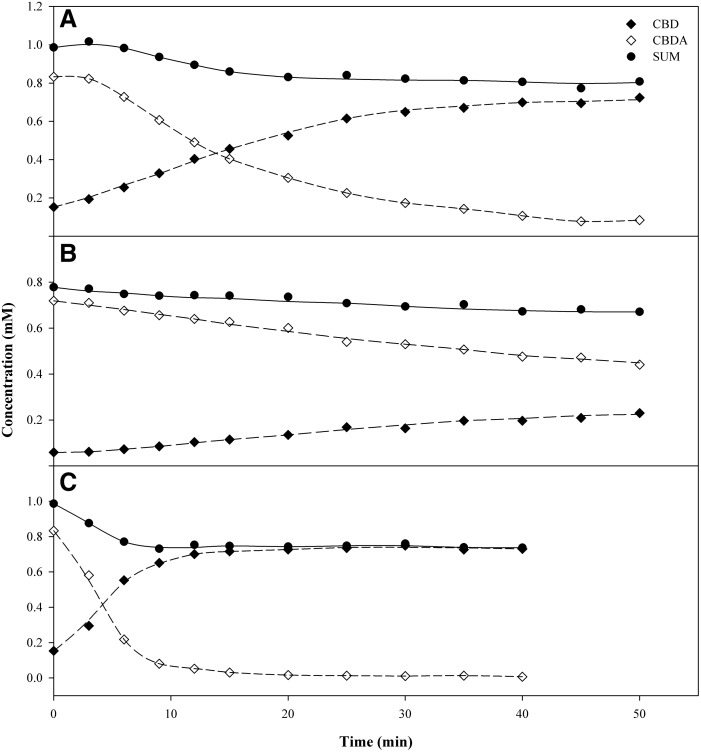
Experimental results for the decarboxylation of **(A)** CBDA in extracts at 110°C; **(B)** pure CBDA reference standard at 110°C; **(C)** CBDA in extracts at 130°C.

The experimental results for the decarboxylation of CBDA are shown in Figures 5 and 6. In this case, the mass balance is not as clear as the THCA-A data. In both 110°C and 130°C, the sum of the molar concentration of CBDA and CBD diminished as the time and temperature of the experiments increase. This indicates more complex chemistry than the stoichiometric conversion of CBDA→CBD. It might also be an indication of compound evaporation under vacuum condition or unidentified products produced at the higher temperature.

To elucidate the possible matrix effect, pure CBDA reference standard isolated in-house was studied. This is the first time any such matrix effects have been investigated. Vials containing 1.0 mg of pure CBDA were simultaneously decarboxylated in the vacuum oven with the extracts at 110°C over a range of time. Three milliliters of acetonitrile:methanol (80:20) was added to each vial to form a concentration of 333.33 μg/mL solution before the UHPSFC/PDA-MS analysis. The total molar concentrations of the acidic and neutral products were measured as a function of time and temperature, and the results are given in Figure 6B. The sum of the molar concentration of *pure* CBDA and its decarboxylation product CBD showed a 14% decrease compared to 18% for the extract. The results are given in Table 1.

#### Cannabigerolic acid

**FIG. 7.**
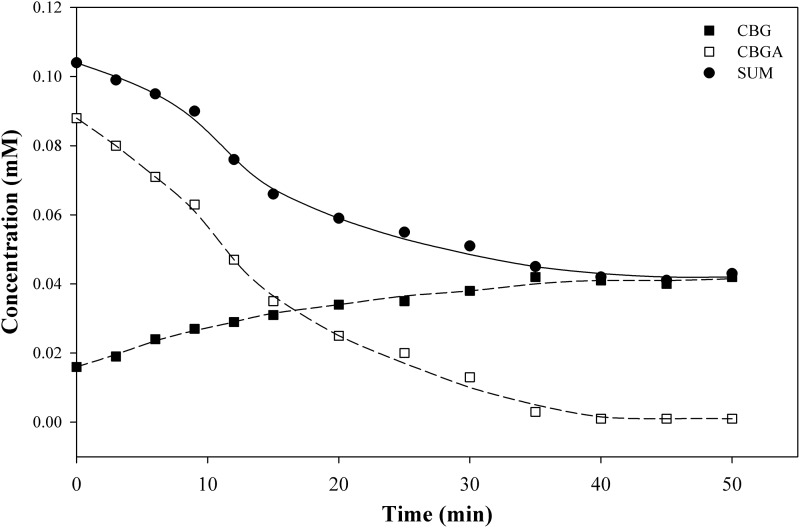
Experimental results for CBG and CBGA at 110°C.

Similar results were obtained for the decarboxylation of CBGA, as shown in Figure 7 and Table 1. In this case, the results are difficult to interpret because of the low concentrations (<0.1 mM) compared to THCA-A and CBD (<1 mM). Despite the low concentrations, the results are very similar to those of CBDA (Figs. 5 and 6).

### Kinetic analyses

To study the decarboxylation reaction, it is important to consider not only the chemical properties of the acidic cannabinoids but also the conditions under which the reaction occurs. The relationship between the rate of the decarboxylation reaction, ${ \frac { d \left[ C \right] }  { dt } }$, and the concentrations of the acidic cannabinoids, $\left[ C \right] ,$ can be expressed by Eq. (1) or the alternative Eq. (2):

\begin{align*}
 { \frac { d \left[ C \right] }  { dt } } = - k \left[ C \right] \tag { 1 } 
\end{align*}

\begin{align*}
ln { \frac { { { [ C ] } _0 } }  { { { [ C ] } _t } } } = kt \tag { 2 } 
\end{align*}

where *k* presents the rate constant, and ${ [ C ] _0}$ and ${ [ C ] _t}$ are the concentrations of reactants at time *0* and *t* min, respectively. The activation energy, *E_A_*, which indicates the minimum energy for the reaction to occur, can be determined from the temperature dependence of the rate constants by the so-called Arrhenius equation, Eq. (3):

\begin{align*}
\ln k = \ln { k_0 } - { \frac { { E_A } }  { RT } } \tag { 3 } 
\end{align*}

where *k_0_* is the frequency factor, and *R* is the gas constant.

Perrotin-Brunel et al.^11^ proposed that the decarboxylation of THCA-A was a direct acid catalyzed keto–enol reaction. The kinetics was first order and the catalyst was a naturally occurring acid in the plant. The results for THCA-A indicated an activation energy, *E_A_*, of 84.8 kJ/mol with a *k_0_* value of 3.7×10^8^ sec^−1^.

**FIG. 8.**
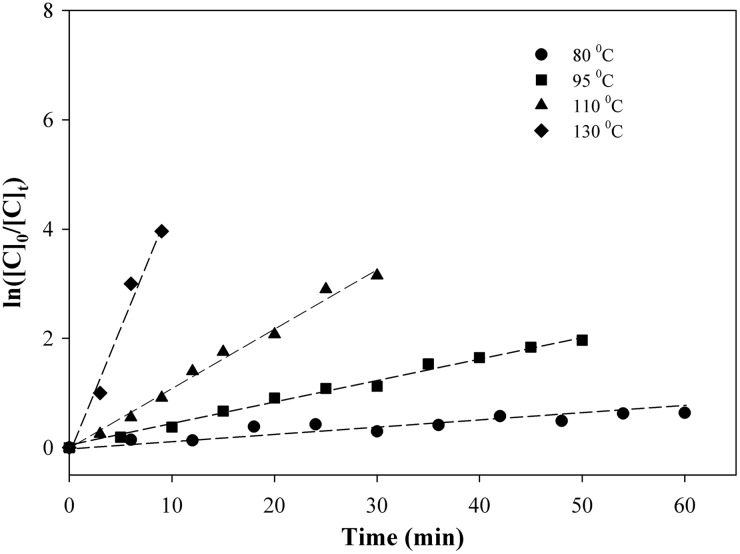
Kinetic results for THCA-A over a range of time and temperature.

The results from the current study are shown in Figure 8 for THCA-A. First-order kinetics is indicated by a logarithmic relationship between the acid concentration and time at a fixed temperature. Figure 8 indicates that first-order kinetics was observed at 80°C, 95°C over the full-time scale, and 110°C and 130°C up to the time when decarboxylation was complete. However, at higher temperature, such as 145°C, the reaction rate was high and the reaction order was difficult to determine. The slopes of the linear plots for 80°C, 95°C and 110°C gave a first-order rate constant for the reaction [from Eq. (2)] and the temperature dependence of these rate constants indicated an *E_A_* value of 88 kJ/mol with a *k_0_* of 8.7×10^8^ sec^−1^ calculated from the Eq. (3).

Similar kinetic analyses were carried out for the three acids used in this study. The first-order rate constants were measured for 80°C, 95°C, and 110°C. The temperature dependence of the rate constants was used to calculate an *E_A_* value for each acid. The results are shown in Table 2. The rate constants for THCA-A were always approximately twice those of CBDA or CBGA, which were nearly identical.

**Table 2.** **Rate Constants, *k*×10^3^ (sec^−1^), and Activation Energetics, *E_A_*, for the Decarboxylation of the Acidic Cannabinoids**

	Rate constants *k*×10^3^ (sec^−1^)			
Reactant	80°C	95°C	110°C	Activation energy *E_A_* (kJ/mol)
Extracts				
THCA-A	0.18	0.66	1.83	88 (84^a^)
CBDA	0.05	0.27	0.83	112
CBGA	0.06	0.25	1.00	109
Pure Standard				
CBDA			0.16	

^a^ Literature value.^11^

## Conclusions

The increasing medical applications of cannabinoids other than Δ^9^-THC demand further investigation of these components and their generation from the acidic precursors that are enzymatically produced in the cannabis plants. This study represents a comprehensive use of UHPSFC/PDA-MS for the analysis of neutral and acidic cannabinoids in determination of the kinetics of phytocannabinoids in cannabis extracts. It also reports the first investigation of the decarboxylation kinetics of all three acid precursors of Δ^9^-THC, CBD, and CBG. UHPSFC has proven to be an excellent separation and quantitation technique for the nine cannabinoids investigated in the current study. The acids and neutrals could be detected in the same experiment without prior decarboxylation or derivatization. UHPSFC allowed the quantitative determination of the concentrations of acids and their decarboxylation products over a range of temperature and time. The variation of acid concentration over times allowed the determination of first-order rate constants. Variation of the rate constants with temperature yielded values for the energy of reaction.

The rate constants for the decarboxylation of THCA-A were higher than those of the other two acids, CBDA and CBGA. The decarboxylation reaction for THCA-A was essentially stoichiometric with no side reactions. In particular, no CBN (a common oxidation by-product) was observed under the experimental conditions. The decarboxylation reactions for CBDA and CBGA were more complex with undetermined side reactions accounting for a loss of 18% (CBDA) to 53% (CBGA) for the extracts. Further study is necessary and will be conducted to provide better understanding of these two acids.

## Supplementary Material

Supplemental data

## Abbreviations Used

ABPR: active back pressure regulator
CBD: cannabidiol
CBDA: cannabidiolic acid
CBG: cannabigerol
CBGA: cannabigerolic acid
CBN: cannabinol
GC: gas chromatography
HPLC/DAD: liquid chromatography-diodide detection
LC: liquid chromatography
MS: mass spectrometry
PDA: photodiode array
SFC: supercritical fluid chromatography
THC: tetrahydrocannabinol
THCA-A: tetrahydrocannabinolic acid-A
THCV: tetrahydrocannabivarin
UHPSFC: ultra-high-performance supercritical fluid chromatography
