# Supplementary material for: Decarboxylation Study of Acidic Cannabinoids: A Novel Approach Using Ultra-High-Performance Supercritical Fluid Chromatography/Photodiode Array-Mass Spectrometry
Source: Cannabis Cannabinoid Res. 2016 Dec 1;1(1):262–71. doi: 10.1089/can.2016.0020 (PMC5549281; doi:10.1089/can.2016.0020)
Supplement: Supplemental data [file Supp_Table1.pdf]

Supplementary Data

Supplementary Table S1. Decarboxylation Studies Over a Range of Temperature and Time

Time (min)	CBD (mM)	CBDA (mM)	Δ^9-THC (mM)	THCA-A (mM)	CBG (mM)	CBGA (mM)
80°C						
0	0.153	0.833	0.284	0.421	0.016	0.088
6	0.165	0.818	0.279	0.299	0.016	0.076
12	0.200	0.828	0.356	0.370	0.017	0.079
18	0.233	0.744	0.386	0.268	0.016	0.079
24	0.242	0.744	0.424	0.257	0.016	0.079
30	0.222	0.807	0.390	0.313	0.016	0.082
36	0.243	0.779	0.427	0.279	0.017	0.077
42	0.250	0.716	0.444	0.237	0.018	0.071
48	0.224	0.770	0.406	0.280	0.018	0.079
54	0.249	0.723	0.448	0.226	0.016	0.076
60	0.261	0.709	0.453	0.223	0.018	0.074
95°C						
0	0.153	0.833	0.284	0.421	0.088	0.088
5	0.192	0.844	0.348	0.348	0.081	0.081
10	0.226	0.765	0.404	0.289	0.076	0.076
15	0.272	0.720	0.464	0.216	0.071	0.071
20	0.308	0.646	0.499	0.160	0.065	0.065
25	0.346	0.606	0.547	0.129	0.065	0.065
30	0.360	0.597	0.550	0.137	0.060	0.060
35	0.409	0.501	0.583	0.091	0.053	0.053
40	0.433	0.469	0.589	0.081	0.050	0.050
45	0.456	0.461	0.604	0.067	0.048	0.048
50	0.495	0.386	0.625	0.059	0.038	0.038
110°C						
0	0.153	0.833	0.284	0.421	0.016	0.088
3	0.194	0.823	0.345	0.373	0.019	0.080
6	0.255	0.728	0.436	0.268	0.024	0.071
9	0.329	0.607	0.508	0.169	0.027	0.063
12	0.404	0.491	0.565	0.104	0.029	0.047
15	0.456	0.404	0.585	0.073	0.031	0.035
20	0.526	0.305	0.621	0.053	0.034	0.025
25	0.615	0.226	0.640	0.021	0.035	0.020
30	0.649	0.174	0.637	0.018	0.038	0.013
35	0.671	0.143	0.638	0.017	0.042	0.003
40	0.699	0.107	0.640	0.014	0.041	0.001
45	0.695	0.078	0.638	0.011	0.040	0.001
50	0.724	0.084	0.639	0.010	0.042	0.001
130°C						
0	0.153	0.833	0.284	0.421	0.016	0.088
3	0.295	0.581	0.469	0.155	0.026	0.057
6	0.553	0.218	0.607	0.021	0.035	0.023
9	0.651	0.080	0.618	0.008	0.042	0.007
12	0.701	0.052	0.628	0.005	0.046	0.003
15	0.716	0.031	0.618	0.004	0.047	0.003
20	0.727	0.016	0.638	0.001	0.048	0.001
25	0.735	0.013	0.638	0.000	0.047	0.001
30	0.749	0.011	0.639	0.000	0.047	0.001
35	0.726	0.013	0.635	0.000	0.047	0.001
40	0.730	0.007	0.631	0.000	0.047	0.001
145°C						
0	0.153	0.833	0.284	0.421	0.016	0.088
3	0.553	0.179	0.564	0.020	0.034	0.018
6	0.714	0.049	0.620	0.007	0.046	0.002
9	0.744	0.017	0.641	0.005	0.052	0.001
12	0.746	0.010	0.614	0.001	0.052	0.001
15	0.752	0.006	0.630	0.001	0.051	0.001
20	0.748	0.005	0.632	0.001	0.050	0.001
25	0.727	0.001	0.630	0.001	0.047	0.001
30	0.714	0.006	0.622	0.001	0.046	0.001

CBD, cannabidiol; CBDA, cannabidiolic acid; CBG, cannabigerol; CBGA, cannabigerolic acid; THC, tetrahydrocannabinol; THCA-A, tetrahydrocannabinolic acid-A.
